# Epidemiology of Functional Abdominal Bloating and Its Impact on Health Related Quality of Life: Male-Female Stratified Propensity Score Analysis in a Population Based Survey in Mainland China

**DOI:** 10.1371/journal.pone.0102320

**Published:** 2014-07-18

**Authors:** Meijing Wu, Yanfang Zhao, Rui Wang, Wenxin Zheng, Xiaojing Guo, Shunquan Wu, Xiuqiang Ma, Jia He

**Affiliations:** 1 Department of Health Statistics, Second Military Medical University, Shanghai, China; 2 Department of Biochemistry and Molecular biology, Bloomberg School of Public Health, Johns Hopkins University, Baltimore, Maryland, United States of America; Iran University of Medical Sciences, Iran, Islamic Republic Of; Baradaran

## Abstract

**Background:**

The epidemiology of Functional abdominal bloating (FAB) and its impact on health-related quality of life (HRQoL) in Chinese people remains unclear.

**Methods:**

Randomised, stratified, multi-stage sampling methodology was used to select a representative sample of the general population from five cities in China (n = 16,078). All respondents completed the modified Rome II questionnaire; 20% were asked to complete the 36-item Short Form (SF-36). The associated factors of FAB were analyzed. The effects of FAB on HRQoL were estimated with gender stratification using propensity score techniques in 20% subsample.

**Results:**

Overall, 643 individuals (4.00%) had FAB and it was more prevalent in males than in females (4.87% vs. 3.04%, *P*<0.001). For males, self-reported history of dyspepsia was most strongly associated with FAB (OR = 2.78; 95% CI: 1.59, 4.72). However, the most strongly associated factor was self-reported health status for females (moderate health vs. good health: OR = 2.06, 95% CI: 1.07, 3.96. *P* = 0.030; poor health vs. good health: OR = 5.71, 95% CI: 2.06, 15.09). Concerning HRQoL, FAB was found to be related to two domains: role limitation due to physical problems (*P* = 0.030) and bodily pain (*P*<0.001) in females. While, in males, there were significant differences in multiple domains between those with and without FAB.

**Conclusion:**

The prevalence of FAB in China was lower than previous reports. Males who had ever been diagnosed with dyspepsia and females who were in a poor self-reported health status were correlated with a higher prevalence of FAB. FAB affected only physical health in females, but impaired both physical and mental health in males.

## Introduction

Functional abdominal bloating (FAB) is a common symptom that affects 10–30% of the human population in western countries. [1] It comprises a group of functional bowel disorders which are dominated by a feeling of abdominal fullness or bloating and without sufficient criteria for another functional gastrointestinal disorder. [1] FAB is usually absent on awakening and worsens throughout the day. It may be intermittent and persists over several days. [1], [2] FAB could arise without any predisposing factors and is unlikely to be completely resolved with medication and lifestyle modification. [1] Surveys indicated that 10%–30% of them experienced bloating often, frequently, or greater than a quarter of the time. [2], [3] However, less than 10% of FAB patients had ever seen doctors as reported in Western and Asia countries. [3], [4] Despite that bloating being a common symptom of several functional gastrointestinal disorders (FGIDs), the Rome classification includes FAB as an independent entity. [5] However, several studies have been conducted to determine the prevalence, characteristics and impact of bloating in patients with some FGIDs, while few large population-based studies on FAB have been carried out in either Western or Asia countries. In China, the prevalence reported in a few studies varied from 8.66% to 11.0% according to the Rome II criteria. [4], [6], [7] The epidemiology of FAB and its effects on Chinese people remain unclear. Furthermore, there are limited data on the impact of FAB on health related quality of life (HRQoL). Only one study in US had mentioned that FAB did not lower HRQoL scores through one-way analysis of variance; [8] however, the confounding factors were not controlled, which might result in biased conclusion. The impact of FAB on HRQoL in Chinese people remains unknown.

For more than two decades, propensity score has been proved to be a more effective means of dealing with large numbers of confounding covariates in observational studies. To our knowledge, a growing body of literature applied propensity score analysis in revealing the association between certain medical condition and HRQoL. [9]–[12] In this research, we evaluated the prevalence characteristics and the associated factors of FAB, as defined by Rome II, in a large representative population and estimated its impact on HRQoL in mainland China using propensity score techniques to achieve balanced distribution of measured covariates between those with and without FAB.

## Materials and Methods

As a part of the large survey of the Systematic Investigation of Gastrointestinal Diseases in China (SILC), the main methods have been described in detail elsewhere, [13] and are summarized here.

### Study design and population

A total of 18,000 residents aged 18–80 years were sampled from 22 residential areas in five cities of China: Shanghai, Beijing, Xi'an, Wuhan and Guangzhou, using a randomized stratified multiple-stage sampling method, with 3,600 residents in each city after stratification by the overall age and sex distribution for each city. Each individual selected was asked to complete a general information questionnaire and the Chinese version of the modified Rome II questionnaire.

The self-completed general information questionnaire was used to collect information on resident region, gender, age, weight, height, marital status, educational level, current job, monthly family income, tobacco use, alcohol use and frequency of activities. Chronic diseases were also recorded. Respondents were asked whether they had been diagnosed by physicians with the following chronic diseases: gastroesophageal reflux disease, dyspepsia, liver disease, hypertension, myocardial infarction, chronic bronchitis, rheumatoid arthritis, osteoarthritis, diabetes etc. There were 51 covariates in total.

The validated Chinese version of the Rome II questionnaire was used to determine the presence of FAB. FAB was defined in accordance with the modified Rome II criteria: a recurrent sensation of abdominal distention that may or may not be associated with measurable distention, but is not part of another functional bowel or gastroduodenal disorder for at least 12 weeks, and onset ≥6 months prior to diagnosis. [14]

A random subsample of 20% of the total sample from each region was asked to complete the Chinese version of the 36-item Short Form (SF-36) to measure HRQoL. SF-36 consists of eight domains: physical function, role limitations due to physical problems (role physical), bodily pain, general health, vitality, social function, role limitations due to emotional problems (role emotional) and mental health. Its reliability and validity have been tested. [13], [15]

### Data collection and response rate

The field work was conducted from April 2007 to January 2008. The questionnaires were self-administered, with trained and supervised facilitators available to explain any questions that respondents were unclear about. Of the 18,000 residents, 16,091 respondents completed the questionnaires with a response rate of 89.40%. Of them, 16,078 were suitable for analysis. A total of 3,219 respondents were randomly selected to complete the SF-36, and analyses were conducted on 3214 respondents. Those data excluded from the analysis were because of the logical errors or insufficient completion of questionnaire. [13], [16]

### Ethical considerations

The study was approved by the Ethics Committee of the Second Military Medical University, Shanghai, China. All participants gave their informed consents to participate in the study and were free to discontinue their participation at any time.

### Statistical analysis

Chi-square test was used to compare the groups with and without FAB. The Cochran-Armitage test was used to detect trends. Generalized Boosted Models was carried out to detect the associated factors of FAB. Odds ratios (ORs) and 95% confidence intervals (CIs) were calculated using multivariate logistic regression. Effects of FAB on the eight dimensions of HRQoL were analyzed by propensity score techniques, with stratification to capture the possible male-female differences. Results from traditional epidemiologic analyses (generalized linear regressions) were also presented. All of the above hypothesis tests were two-sided and a *P*-value of 0.05 or less was considered to indicate statistical significance.

#### Propensity score analysis

The “exposure” variable was FAB versus none. The outcome was defined as eight domains of HRQoL. All the information in general information questionnaire were considered potentially confounding covariates included in the analyses. All 51 covariates were modeled as either categorical or binary factors. List-wise deletion method was conducted to remove those with missing value, thus 3,179 respondents was finally included in the analysis. Generalized Boosted Models was used to estimate the propensity scores. Two matching methods (optimal 1:1 matching and full matching), subclassification (5 subclasses) and weighting were used for propensity score applications. Then the average standardized absolute mean distance was chose as a measure of covariate balance. Our decision criteria identified the techniques that yielded the smallest average standardized absolute mean distance, and a standardized bias of 0.1 or greater may be of concern for each covariate. [17]–[19] The final outcome models (generalized linear regression models on matched data sets or weighted generalized linear regression models) were adjusted for those covariates which remained imbalance after each propensity score technique and those with relative influence bigger than 2% to account for residual confounding. We also presented results from generalized linear regressions without propensity score adjusted to be compared.

#### Software

All statistical analyses were conducted in the R language. For propensity score analysis, we use two software packages written for R: MatchIt [20] and Twang [21]. Twang, which calls the ‘Generalized Boosted Models’ package is used to estimate propensity score and weighting. MatchIt is used for the propensity score application methods involving matching (optimal 1:1 and full) and sub-classification. For matching, MatchIt calls the R ‘optmatch’ package. [19], [22]

## Results and Discussion

### Prevalence of FAB

Of the 16,078 respondents, a total of 643 participants (4.00%) were classified as having FAB according to the Rome II criteria (Table 1). FAB was more prevalent in men than in women (4.87% vs. 3.04%, χ^2^ = 35.04, *P*<0.001). The prevalence of FAB rose with increasing age in the combined sample (trend test: χ^2^ = 7.56, *P* = 0.006), while there were no such trends in either females or males (trend test for females: χ^2^ = 2.97, *P* = 0.085; trend test for males: χ^2^ = 3.90, *P* = 0.048). There were no significant differences between urban and rural area (3.80% vs. 4.20%, χ^2^ = 1.62, *P* = 0.203). The prevalence of FAB in Shanghai, Beijing, Xi'an, Wuhan, and Guangzhou was 3.33%, 4.58%, 4.45%, 5.08% and 2.52%, respectively, and it varied significantly among the five study regions (χ^2^ = 36.32, *P*<0.001).

**Table 1:** The prevalence of functional abdominal bloating from a cross sectional study in five cities, china, 2007–2008(n = 16,078).

	With FAB	Without FAB	Prevalence (%)	P-value	
Gender**				<0.001	
Female	234	7454	3.04		
Male	409	7981	4.87		
Age(years)* a				0.006	
<30	138	3542	3.75		
30∼40	140	3535	3.81		
40∼50	137	3675	3.59		
50∼60	100	2368	4.05		
60∼70	77	1426	5.12		
> = 70	51	889	5.43		
Area				0.203	
Urban	307	7765	3.80		
Rural	336	7670	4.20		
Site**				<0.001	
Shanghai	105	3046	3.33		
Beijing	145	3023	4.58		
Wu han	146	3137	4.45		
Xi an	166	3100	5.08		
Guangzhou	81	3129	2.52		
All	643	15435	4.00		

*P<0.05, Chi-squared Test.

**P<0.01.

a Cochran–Armitage test.

### Associated factors

The relative influence of each confounding variable was obtained from the Generalized Boosted Models, which indicated a variable's contribution to estimate the predicted probabilities of FAB from the 16,078 respondents. The higher the contribution, the more important the confounding variable was for prediction, while irrelevant ones had a minimal effect. [23], [24] Thus, the variables with non-zero relative influence could be considered as the potential associated factors for FAB. Then multivariate logistic regression was carried out to detect statistical significance of those factors.

Relative influences of the top 10 potential associated factors for FAB were presented in table 2. For males, self-reported history of dyspepsia was most strongly associated with FAB. There was significant difference in the prevalence of FAB between those having ever been diagnosed with dyspepsia and those never (13.57% vs. 4.21%, OR = 2.78; 95% CI: 1.59, 4.72). The other two associated factors were self-reported history of gastritis (OR = 1.74, 95% CI: 1.04, 2.83), and self-reported history of chronic hoarseness (OR = 4.15, 95% CI: 1.44, 10.99) according to the results of multivariate logistic regression. However, the most strongly associated factor for FAB was self-reported health status for females. Those reported in moderate or poor health were having a higher prevalence of FAB than those in good health (moderate health vs. good health: 7.34% vs. 3.51%, OR = 2.06, 95% CI: 1.07, 3.96; poor health vs. good health: 11.83% vs. 3.51%, OR = 5.71, 95% CI: 2.06, 15.09). Except for this, only self-reported history of gastritis (OR = 2.00, 95% CI: 1.05, 3.67) was the associated factor with statistically significance for females.

**Table 2:** Relative influence of the top10 potential associated factors for functional abdominal bloating using generalized boosted models, a cross sectional study in five cities, china, 2007–2008 (n = 16,078).

Females		Males	
Variable	Relative influence(%)	Variable	Relative influence(%)
Self-reported health status	16.62	Self-reported history of dyspepsia	68.63
Self-reported history of dyspepsia	10.75	Body Mass Index	4.68
Self-reported history of Irritable bowel syndrome	4.24	City of residence	4.11
Self-reported history of Rheumatoid arthritis	4.02	Self-reported mental status	3.44
Self-reported social activity	3.90	Self-reported health status	3.41
Self-reported work pressure	3.90	Self-reported ability of daily activity	2.70
Self-reported ability of work	3.63	Self-reported history of gastritis	2.03
Self-reported mental status	3.63	Marital status	1.63
Self-reported ability of daily activity	3.58	Self-reported history of chronic pharyngitis	1.32
Self-reported history of gastritis	3.01	Self-reported history of chronic hoarseness	1.28

### Covariate balance

Prior to running the final outcome regressions, we evaluated all 4 propensity score techniques in their ability to balance the measured covariates between our exposure and comparison groups using the aforementioned decision criteria (Table 3). In general, the optimal 1:1 matching performed the best, generating the lowest average standardized absolute mean distance in all three datasets, but only 65 pairs in females, 110 pairs in males and together 175 pairs in the combined subsample were matched, which lost 91%, 87% and 89% information, respectively. Due to the drastic reduction in sample sizes, the optimal 1:1 matching might not be an appropriate method to estimate the effect of FAB. Thus we make the final conclusion through the results of full matching for the females, and weighting for males and the whole subsample.

**Table 3:** Average standardized absolute mean distance before and after propensity score adjustment by 4 different applications in 20% of the total respondents, a cross sectional study in five cities, china, 2007–2008 (n = 3,179).

	Pre-PS adjustment	Post-PS adjustment			
		1:1 matching	Full matching	subclassification	Weighting
Females	0.13	0.04	0.04	0.11	0.08
Males	0.11	0.04	0.11	0.10	0.04
All	0.12	0.03	0.12	0.09	0.04

For the combined subsample, those who had FAB were different from comparison individuals on many covariates. Compared with the comparison groups, a higher percentage of FAB subjects were found in moderate or poor self-reported health and mental status, or with moderate or poor ability of daily activity and work (Table 4). Those who suffered from FAB tended to report more history of dyspepsia, hypertension, cerebrovascular disease and osteoarthritis, etc, than comparison individuals (Table 5). The application of the propensity score corrected for most of the imbalances, as evidenced by the decrease in measured covariate standardized bias below 0.10 for most of the covariates after propensity score adjustment. Characteristics of the entire baseline and disease history covariates and their standardized bias before and after propensity score adjusted could be seen in Appendix S1. Across males, females, and the combined subsample, the FAB subjects and comparison individuals do not appear to have markedly different baseline or disease history levels, thus we did not provide the comparison results separated by females and males here.

**Table 4:** Characteristics of baseline covariates with *p*<0.05 before adjusted and standardized bias before and after PS adjusted using weighting in 20% of the total respondents, a cross sectional study in five cities, china, 2007–2008 (n = 3,179).

		With FAB (%, n = 175)	Without FAB (%, n = 3004)	Chi square	*P* value	Standardized bias before PS adjusted	Standardized bias after PS adjusted
Gender	Female	65(37.14)	1453(48.37)	8.35*	0.004	0.23	0.06
	Male	110(62.86)	1551(51.63)			0.23	0.06
Age(years)	<30	34(19.43)	705(23.47)	11.09*	0.050	0.10	0.01
	30∼40	35(20.00)	693(23.07)			0.08	0.01
	40∼50	43(24.57)	705(23.47)			0.03	0.01
	50∼60	22(12.57)	460(15.31)			0.08	0.12
	60∼70	26(14.86)	281(9.35)			0.15	0.08
	> = 70	15(8.57)	160(5.33)			0.12	0.02
Education	Primary school or lower	44(25.14)	523(17.41)	8.16*	0.017	0.18	0.09
	Secondary/high school	93(53.14)	1880(62.58)			0.19	0.11
	University or higher	38(21.71)	601(20.01)			0.04	0.04
Alcohol consumption	No	152(86.86)	2350(78.23)	7.34*	0.007	0.25	0.07
	Yes	23(13.14)	654(21.77)			0.25	0.07
Self-reported health status	Good	61(34.86)	1679(55.89)	35.79**	<0.001	0.44	0.08
	Moderate	92(52.57)	1161(38.65)			0.28	0.06
	Poor	22(12.57)	164(5.46)			0.21	0.03
Self-reported ability of daily activity	Good	96(54.86)	2011(66.94)	13.31*	0.001	0.24	0.02
	Moderate	69(39.43)	912(30.36)			0.19	0.02
	Poor	10(5.71)	81(2.70)			0.13	0
Self-reported ability of work	Good	97(55.43)	2019(67.21)	15.23**	<0.001	0.24	0.03
	Moderate	65(37.14)	889(29.59)			0.16	0.01
	Poor	13(7.43)	96(3.20)			0.16	0.04
Self-reported mental status	Good	95(54.29)	2057(68.48)	15.53**	<0.001	0.28	0.06
	Moderate	70(40.00)	845(28.13)			0.24	0.07
	Poor	10(5.71)	102(3.40)			0.1	0.02

*P<0.05.

**P<0.001.

**Table 5:** Characteristics of disease history with *p*<0.05 before adjusted and standardized bias before and after PS adjusted using weighting in 20% of the total respondents, a cross sectional study in five cities, china, 2007–2008 (n = 3,179).

		With FAB (%, n = 175)	Without FAB (%, n = 3004)	Chi square	*P* value	Standardized bias before PS adjusted	Standardized bias after PS adjusted	
Self-reported history of dyspepsia	No	139(79.43)	2800(93.21)	44.99	<0.001	0.34	0.07	
	Yes	36(20.57)	204(6.79)			0.34	0.07	
Self-reported history of liver disease	No	160(91.43)	2873(95.64)	6.69	0.010	0.15	0.08	
	Yes	15(8.57)	131(4.36)			0.15	0.08	
Self-reported history of hypertension	No	139(79.43)	2628(87.48)	9.51	0.002	0.2	0.07	
	Yes	36(20.57)	376(12.52)			0.2	0.07	
Self-reported history of cerebrovascular disease	No	164(93.71)	2923(97.30)	7.58	0.006	0.15	0.02	
	Yes	11(6.29)	81(2.70)			0.15	0.02	
Self-reported history of chronic bronchitis	No	160(91.43)	2872(95.61)	6.54	0.011	0.15	0.06	
	Yes	15(8.57)	132(4.39)			0.15	0.06	
Self-reported history of rheumatoid arthritis	No	157(89.71)	2872(95.61)	12.77	<0.001	0.19	0.05	
	Yes	18(10.29)	132(4.39)			0.19	0.05	
Self-reported history of osteoarthritis	No	160(91.43)	2909(96.84)	14.48	<0.001	0.19	0.07	
	Yes	15(8.57)	95(3.16)			0.19	0.07	
Self-reported history of gastritis	No	125(71.43)	2637(87.78)	38.81	<0.001	0.36	0.07	
	Yes	50(28.57)	367(12.22)			0.36	0.07	
Self-reported history of gallbladder disease	No	155(88.57)	2864(95.34)	15.85	<0.001	0.21	0.06	
	Yes	20(11.43)	140(4.66)			0.21	0.06	
Self-reported history of Irritable Bowel Syndrome	No	172(98.29)	2999(99.83)	.	0.008	0.12	0.11	
	Yes	3(1.71)	5(0.17)			0.12	0.11	
Self-reported history of chronic pharyngitis	No	144(82.29)	2716(90.41)	12.10	<0.001	0.21	0.03	
	Yes	31(17.71)	288(9.59)			0.21	0.03	
Self-reported history of chronic hoarseness	No	166(94.86)	2975(99.03)	21.03	<0.001	0.19	0.09	
	Yes	9(5.14)	29(0.97)		<0.001	0.19	0.09	
Self-reported history of chronic cough	No	162(92.57)	2959(98.50)	29.24	<0.001	0.23	0.07	
	Yes	13(7.43)	45(1.50)			0.23	0.07	
Self-reported history of eczema	No	166(94.86)	2933(97.64)	4.14	0.042	0.13	0.04	
	Yes	9(5.14)	71(2.36)			0.13	0.04	

### Propensity score adjustment

The final β-estimate and its 95% confidence interval from the propensity score adjusted regression models are presented in table 6 and table 7. After adjusting for all the factors, FAB was found to be related to role physical (β-estimate = −6.88; 95% Cl: −12.99,−0.77), bodily pain (β-estimate = −6.12; 95% Confidence Interval: −10.29, −1.95) domains in females after applying full matching. The application of weighting showed that 7 from all 8 SF-36 domains, except physical function, were significant different between those males with and without FAB (*P*<0.05), while all the 8 SF-36 domains were in significant correlation with FAB for the combined sample (*P*<0.05).

**Table 6:** Effect of FAB on health related quality of life by males and females separately, with propensity score techniques to control the confounding factors in 20% of the total respondents, a cross sectional study in five cities, china, 2007–2008 (n = 3,179).

	SF-36 domains^a^		Logistic regression		Propensity score adjustment by Full matching		Propensity score adjustment by Weighting by the odds
		β-estimate	95% confidence interval	β-estimate	95% confidence interval	β-estimate	95% confidence interval
Females	Physical function	−1.72	−3.94, 0.50	−1.35	−3.77, 1.08		
	Role physical	−6.49*	−12.55, −0.43	−6.88*	−12.99, −0.77		
	Bodily pain	−6.09***	−10.22, −1.97	−6.12***	−10.29, −1.95		
	General health	−2.01	−5.97, 1.94	−1.88	−5.84, 2.08		
	Vitality	−2.60	−6.46, 1.27	−2.82	−6.70, 1.07		
	Social function	−2.21	−6.08, 1.66	−1.90	−5.78, 1.98		
	Role emotional	−2.08	−8.47, 4.30	−1.46	−7.84, 4.92		
	Mental health	−0.02	−3.56, 3.53	0.38	−3.17, 3.93		
Males	Physical function	−0.94	−3.25, 1.38			−1.10	−2.25, 0.06
	Role physical	−3.46	−8.94, 2.03			−3.67*	−6.68, −0.66
	Bodily pain	−3.09	−6.71, 0.54			−2.64*	−4.59, −0.70
	General health	−4.14*	−7.43, −0.85			−4.13***	−5.83, −2.44
	Vitality	−4.27*	−7.55, −0.98			−4.29***	−5.95, −2.62
	Social function	−3.44*	−6.42, −0.46			−3.38***	−4.94, −1.81
	Role emotional	−4.94	−10.57, 0.7			−4.63***	−7.77, −1.49
	Mental health	−3.02*	−5.88, −0.16			−2.55***	−3.97, −1.14

Role emotional refers to role limitations due to emotional problems; Role physical, refers to role limitations due to physical problems;

*P<0.05, **P<0.01, ***P<0.001;

a The brief self-administered questionnaire SF-36 was used to measure health related quality of life. Item scores for each SF-36 domains were coded, summed and transformed to a scale from 0 (worst possible health state) to 100 (best possible health state).

**Table 7:** Effect of FAB on health related quality of life in the combined subsample, with propensity score techniques to control the confounding factors in 20% of the total respondents, a cross sectional study in five cities, china, 2007–2008 (n = 3,179).

	SF-36 domains^a^	Logistic regression		Propensity score adjustment by Weighting by the odds	
		β-estimate	95% confidence interval	β-estimate	95% confidence interval
the Combined Subsample	Physical function	−0.54	−3.60, 2.52	−0.95*	−1.82, −0.09
	Role physical	−4.26*	−8.33, −0.20	−3.37 ***	−5.58, −1.15
	Bodily pain	−3.94***	−6.64, −1.24	−3.84***	−5.22, −2.46
	General health	−3.31*	−5.80, −0.81	−3.13***	−4.36, −1.91
	Vitality	−3.75***	−6.22, −1.28	−3.26***	−4.45, −2.06
	Social function	−2.94*	−5.28, −0.60	−2.50***	−3.65, −1.36
	Role emotional	−3.35	−7.54, 0.83	−3.10*	−5.30, −0.90
	Mental health	−1.69	−3.90, 0.51	−1.38*	−2.42, −0.34

Role emotional refers to role limitations due to emotional problems; Role physical, refers to role limitations due to physical problems;

*P<0.05, **P<0.01, ***P<0.001;

a The brief self-administered questionnaire SF-36 was used to measure health related quality of life. Item scores for each SF-36 domains were coded, summed and transformed to a scale from 0 (worst possible health state) to 100 (best possible health state).

### Traditional adjustment

The traditional generalized linear regression produces results that are different from the propensity score adjusted model. For females, the β estimates comparing role physical and bodily pain domains among those with and without FAB were statistically significant (*P*<0.05). For males, β estimates in general health, vitality, social function, mental health domains were statistically significant (*P*<0.05). For the combined sample, FAB was found to be correlated with five SF-36 domains: role physical, bodily pain, general health, vitality, and social function. The propensity score adjusted analyses are generally preferred because they ensure the balance of covariates between the FAB subjects and comparison groups. [19]

## Discussion

This was the first large, population-based epidemiological study of FAB that included a total population of 16,078 respondents from five regions in China. 4.00% of the respondents have FAB as defined by Rome II in our study. The result was not consistent with previous observations before. In 2001, it was reported that the prevalence of FAB in general population was 15% in China. [25] An investigation in Guangzhou between 1999 and 2000 indicated that the prevalence of FAB was 11.0%. [4] While in our study, the prevalence of FAB in Guangzhou area was 2.52%. The investigation by Guangzhou randomly selected the participants from the people who did physical examination in a hospital, which might introduce sampling bias. In an American householder survey in 1990, 27.3% of 5430 respondents reported to have FAB. [3] The study population in the survey was identified through a mailing list and consisted of only households that had agreed to participate in the survey, making it susceptible to sampling bias. Another study of employees in western South Dakota in 2008 reported that only 7% of subjects met criteria for FAB. [8] In general, the prevalence of FAB in our study is lower than previously reported in both China and Western countries. One reason might be that we used modified Rome II criteria in which FAB onset ≥6 months prior to diagnosis, while the other studies defined the symptom in the preceding 12 months. In addition, many patients with bloating had symptoms of irritable bowel syndrome, functional dyspepsia or constipation as reported. [2] Previous research usually confused FAB with those diseases, which might lead to higher prevalence. The prevalence did not vary between the city and urban areas, while it varied significantly among Shanghai, Beijing, Xi'an, Wuhan, and Guangzhou. Studies on the causes of the regional differences are needed.

In our study, we also found that more men reported having FAB compared with women, and the prevalence rose with increasing age for the total sample, while there was no such trend in separated analysis of females or males. The investigation in Guangzhou indicated that there was no statistically difference between females (11.6%) and males (10.4%), and not among the different age groups. [4] However, in the American householder survey, females were less likely to have FAB (34.3% for males, and 27.3% for females). The prevalence was similar between different age groups for males, but increased with age for females. [3] As mentioned above, these two studies both have sampling bias. It has been reported that recent weight gain would cause symptoms of FAB. [2] Men usually experienced higher level of pressure both in work and life. They spent more time in work and less time in exercise. In addition, they had to drink a lot when attending the frequent work-related event. As a result, men tend to gain weight easily than women, which perhaps causing the difference between men and women in the prevalence of FAB.

A review indicated that recent weight gain, weak abdominal muscles, and lack of exercise might cause FAB. [2] However, in this study, we found that the associated factors of FAB were quite different between females and males, which had never been reported previously. For males, self-reported history of dyspepsia was most strongly associated with FAB. Those having ever been diagnosed with dyspepsia were 2.78 times more likely to develop FAB than those never. The prevalence of functional dyspepsia was between 20% and 40% as reported both in western countries and china. [4] Approximately 50% of the subjects fulfilling modified Rome II criteria for dyspepsia reported bloating. [5] Those who had ever been diagnosed with gastritis or chronic hoarseness were all having a higher prevalence of FAB than those never in males. For females, the most strongly associated factor for FAB was self-reported health status. Those reported in moderate or poor health were 2.06 or 5.71 times more likely to develop FAB than those in good health. Besides, the association between self-reported history of gastritis and FAB in females was similar with males. Studies on the associated factors of FAB are needed.

What we found about the relationship between FAB and HRQoL in this study was different from what had reported before, which mentioned that FAB did not lower HRQoL scores through one-way analysis of variance without confounding factors controlled. For females, we found that FAB had little association with other HROoL domains but role physical and bodily pain, indicating that physical activities, general health, vitality, social function, psychological function and mental health did not impaired by FAB in women subjects. In the other hand, FAB patients might have role limitations due to physical problems, as reported in an American study that the rate for work absenteeism (currently too sick to work or to go to school) was 6.3% for FAB patients. [3] Furthermore, FAB could be a disease associated with bodily pain. According to a review of FAB, the sufferer awakened with a flat abdomen that progressively enlarges as the day went on. Large or heavy meals made it worse as may constipation and the distention was visible. [2] For males, 7 of all 8 HRQoL domains were found relate to male FAB subjects except Physical function, which illustrated that performing physical activities was not significantly limited by FAB. Compared with females, this study showed that FAB impaired not only physical but also mental health in males. It has been reported in two large population surveys that bloating correlated with psychiatric dysfunction: depression, sleeping difficulties, problems of coping, panic disorder, and agoraphobia. [2], [26], [27] For the combined sample, FAB influenced all the HRQoL domains, in which physical function did not find significantly associate with neither female nor male subjects alone.

The strengths of our study are obvious: first, it was the first large, truly population-based study of the epidemiology of FAB in China that used validated questionnaires. The study provides important and generalizable data on the epidemiology of FAB and has the potentials to make a major contribution to the epidemiological understanding of FAB in China. Second, it was the first application of propensity score techniques to adjust the confounding factors in exploring the relationship between FAB and HRQoL. Third, Generalized Boosted Models were used for identifying the potential associated factors of FAB instead of the traditional regression methods. However, the study has a few potential limitations. Only 20% of the sample were evaluated their HRQoL. Another limitation is that we could not balance unobserved or unmeasured covariates. However, this concern over potential unmeasured confounders is common to both propensity score techniques and traditional multivariable regression applied to observational data. [18]

## Conclusions

In conclusion, the prevalence of FAB in China was lower than previous reports. More men reported having FAB compared with women. Men who had ever been diagnosed with dyspepsia and women who were in a poor self-reported health status were correlated with a higher prevalence of FAB than others. FAB affected only physical health in females, but impaired both physical and mental health in males.

## Supporting Information

Appendix S1 **Characteristics of baseline covariates and standardized bias before and after PS adjusted using weighting by the odds in 20% of the total respondents, a cross sectional study in five cities, china, 2007–2008 (n = 3,179).**
(PDF)
