# Supplementary material for: Epidemiology of Functional Abdominal Bloating and Its Impact on Health Related Quality of Life: Male-Female Stratified Propensity Score Analysis in a Population Based Survey in Mainland China
Source: PLoS One. 2014 Jul 18;9(7):e102320. doi: 10.1371/journal.pone.0102320 (PMC4103840; doi:10.1371/journal.pone.0102320)
Supplement: Appendix S1 — Characteristics of baseline covariates and standardized bias before and after PS adjusted using weighting by the odds in 20% of the total respondents, a cross sectional study in five cities, china, 2007–2008 (n = 3,179). (PDF) [file pone.0102320.s001.pdf]

Appendix S1. Characteristics of baseline covariates and standardized bias before and after ps adjusted using weighting by the odds in 20% of the total respondents, a cross sectional study in five cities, china, 2007-2008 (n=3,179)

		With FAB (%, n=175)	Without FAB (%, n=3004)	Chi square	P value	Standardized bias before PS adjusted	Standardized bias after PS adjusted using weighting
Region of residence	Urban	83(47.43)	1482(49.33)	0.24	0.624	0.04	0.06
	Rural	92(52.57)	1522(50.67)			0.04	0.06
Gender	Female	65(37.14)	1453(48.37)	8.35*	0.004	0.23	0.06
	Male	110(62.86)	1551(51.63)			0.23	0.06
Age(years)	<30	34(19.43)	705(23.47)	11.09*	0.050	0.10	0.01
	30~40	35(20.00)	693(23.07)			0.08	0.01
	40~50	43(24.57)	705(23.47)			0.03	0.01
	50~60	22(12.57)	460(15.31)			0.08	0.12
	60~70	26(14.86)	281(9.35)			0.15	0.08
	>=70	15(8.57)	160(5.33)			0.12	0.02
Body Mass Index(kg/m^2)	<18.5	12(6.86)	281(9.35)	1.32	0.517	0.1	0.06
	18.5-23	86(49.14)	1469(48.90)			0	0.02
	>=23	77(44.00)	1254(41.74)			0.05	0.01
Marital status	Married	141(80.57)	2323(77.33)	4.74	0.093	0.08	0.03
	Unmarried	22(12.57)	542(18.04)			0.16	0.05
	Others	12(6.86)	139(4.63)			0.09	0.01
Education	Primary school or lower	44(25.14)	523(17.41)	8.16*	0.017	0.18	0.09
	Secondary/high school	93(53.14)	1880(62.58)			0.19	0.11
	University or higher	38(21.71)	601(20.01)			0.04	0.04
Nation	Han	173(98.86)	2962(98.60)	0.21	0.902	0.02	0.02
	Others	2(1.14)	39(1.30)			0.01	0.01
	Missing	0(0.00)	3(0.10)			0	0
Occupation	Office worker	47(26.86)	856(28.50)	0.22	0.640	0.04	0.04
	Manual worker	128(73.14)	2148(71.50)			0.04	0.04
Monthly family income (Yuan)	<1999	92(52.57)	1532(51.00)	2.29	0.319	0.03	0
	2000~4999	73(41.71)	1200(39.95)			0.04	0.03
	>5000	10(5.71)	272(9.05)			0.14	0.06
Smoking status	Never smoker	133(76.00)	2094(69.71)	5.45	0.065	0.15	0.05
	Current smoker	34(19.43)	813(27.06)			0.19	0.07
	Ex-smoker	8(4.57)	97(3.23)			0.06	0.03
Alcohol consumption	No	152(86.86)	2350(78.23)	7.34*	0.007	0.25	0.07
	Yes	23(13.14)	654(21.77)			0.25	0.07

*P<0.05 ** P<0.001

Appendix S1. (**Continued**) Characteristics of baseline covariates and standardized bias before and after ps adjusted using weighting by the odds in 20% of the total respondents, a cross sectional study in five cities, china, 2007-2008 (n=3,179)

		With FAB (%, n=175)	Without FAB (%, n=3004)	Chi square	P value	Standardized bias before PS adjusted	Standardized bias after PS adjusted using weighting
Frequency of recreational exercise	Never	33(18.86)	489(16.28)	2.18	0.535	0.07	0.04
	Less than weekly	12(6.86)	294(9.79)			0.12	0.14
	At least weekly but less than daily	26(14.86)	460(15.31)			0.01	0.01
	At least daily	104(59.43)	1761(58.62)			0.02	0.03
Self-reported health status	Good	61(34.86)	1679(55.89)	35.79**	<0.001	0.44	0.08
	Moderate	92(52.57)	1161(38.65)			0.28	0.06
	Poor	22(12.57)	164(5.46)			0.21	0.03
Self-reported ability of daily activity	Good	96(54.86)	2011(66.94)	13.31*	0.001	0.24	0.02
	Moderate	69(39.43)	912(30.36)			0.19	0.02
	Poor	10(5.71)	81(2.70)			0.13	0
Self-reported ability of work	Good	97(55.43)	2019(67.21)	15.23**	<0.001	0.24	0.03
	Moderate	65(37.14)	889(29.59)			0.16	0.01
	Poor	13(7.43)	96(3.20)			0.16	0.04
Self-reported life pressure	No/ a little	116(66.29)	2136(71.11)	2.73	0.255	0.1	0
	Moderate	30(17.14)	492(16.38)			0.02	0.02
	Quite a lot/Extreme	29(16.57)	376(12.52)			0.11	0.03
Self-reported work pressure	No/ a little	122(69.71)	2315(77.06)	5.31	0.070	0.16	0.07
	Moderate	31(17.71)	427(14.21)			0.09	0.03
	Quite a lot/Extreme	22(12.57)	262(8.72)			0.12	0.06
Self-reported mental status	Good	95(54.29)	2057(68.48)	15.53**	<0.001	0.28	0.06
	Moderate	70(40.00)	845(28.13)			0.24	0.07
	Poor	10(5.71)	102(3.40)			0.1	0.02
Self-reported social activity	Good	103(58.86)	1992(66.31)	4.46	0.108	0.15	0.01
	Moderate	68(38.86)	938(31.23)			0.16	0.02
	Poor	4(2.29)	74(2.46)			0.01	0.04

*P<0.05 ** P<0.001

Appendix S1. (**Continued**) Characteristics of disease history covariates and standardized bias before and after ps adjusted using weighting by the odds in 20% of the total respondents, a cross sectional study in five cities, china, 2007-2008 (n=3,179)

		With FAB (%, n=175)	Without FAB (%, n=3004)	Chi square	P value	Standardized bias before PS adjusted	Standardized bias after PS adjusted using weighting
Family history of GI disease	No	155(88.57)	2696(89.75)	0.25	0.619	0.04	0.04
	Yes	20(11.43)	308(10.25)			0.04	0.04
Self-reported history of GERD	No	166(94.86)	2935(97.70)	4.47	0.035	0.13	0.02
	Yes	9(5.14)	69(2.30)			0.13	0.02
Self-reported history of dyspepsia	No	139(79.43)	2800(93.21)	44.99**	<0.001	0.34	0.07
	Yes	36(20.57)	204(6.79)			0.34	0.07
Self-reported history of liver disease	No	160(91.43)	2873(95.64)	6.69*	0.010	0.15	0.08
	Yes	15(8.57)	131(4.36)			0.15	0.08
Self-reported history of hypertension	No	139(79.43)	2628(87.48)	9.51*	0.002	0.2	0.07
	Yes	36(20.57)	376(12.52)			0.2	0.07
Self-reported history of Myocardial infarction	No	175(100.00)	2992(99.60)	.	1.000	0	0
	Yes	0(0.00)	12(0.40)			0	0
Self-reported history of Angina	No	170(97.14)	2955(98.37)	0.84	0.358	0.07	0.01
	Yes	5(2.86)	49(1.63)			0.07	0.01
Self-reported history of cerebrovascular disease	No	164(93.71)	2923(97.30)	7.58*	0.006	0.15	0.02
	Yes	11(6.29)	81(2.70)			0.15	0.02
Self-reported history of chronic bronchitis	No	160(91.43)	2872(95.61)	6.54*	0.011	0.15	0.06
	Yes	15(8.57)	132(4.39)			0.15	0.06
Self-reported history of asthma	No	173(98.86)	2965(98.70)	0.00	1.000	0.01	0.06
	Yes	2(1.14)	39(1.30)			0.01	0.06
Self-reported history of kidney disease	No	167(95.43)	2924(97.34)	1.58	0.208	0.09	0.01
	Yes	8(4.57)	80(2.66)			0.09	0.01
Self-reported history of rheumatoid arthritis	No	157(89.71)	2872(95.61)	12.77**	<0.001	0.19	0.05
	Yes	18(10.29)	132(4.39)			0.19	0.05

*P<0.05 ** P<0.001

Appendix S1. (**Continued**) Characteristics of disease history covariates and standardized bias before and after ps adjusted using weighting by the odds in 20% of the total respondents, a cross sectional study in five cities, china, 2007-2008 (n=3,179)

		With FAB (%, n=175)	Without FAB (%, n=3004)	Chi square	P value	Standardized bias before PS adjusted	Standardized bias after PS adjusted using weighting
Self-reported history of osteoarthritis	No	160(91.43)	2909(96.84)	14.48**	<0.001	0.19	0.07
	Yes	15(8.57)	95(3.16)			0.19	0.07
Self-reported history of anxiety disorder	No	174(99.43)	2996(99.73)	.	0.400	0.04	0.01
	Yes	1(0.57)	8(0.27)			0.04	0.01
Self-reported history of depression	No	175(100.00)	2991(99.57)	.	1.000	0	0
	Yes	0(0.00)	13(0.43)			0	0
Self-reported history of abdominal surgery	No	141(80.57)	2525(84.05)	1.48	0.223	0.09	0.03
	Yes	34(19.43)	479(15.95)			0.09	0.03
Self-reported history of diabetes	No	167(95.43)	2909(96.84)	1.05	0.306	0.07	0.01
	Yes	8(4.57)	95(3.16)			0.07	0.01
Self-reported history of chronic leg ulcer	No	175(100.00)	3003(99.97)	.	1.000	0	0
	Yes	0(0.00)	1(0.03)			0	0
Self-reported history of sever visual impairment	No	173(98.86)	2976(99.07)	0.00	1.000	0.02	0.04
	Yes	2(1.14)	28(0.93)			0.02	0.04
Self-reported history of dysphagia	No	174(99.43)	2995(99.70)	.	0.433	0.04	0.08
	Yes	1(0.57)	9(0.30)			0.04	0.08
Self-reported history of gastritis	No	125(71.43)	2637(87.78)	38.81**	<0.001	0.36	0.07
	Yes	50(28.57)	367(12.22)			0.36	0.07
Self-reported history of peptic ulcer	No	163(93.14)	2868(95.47)	2.02	0.155	0.09	0.01
	Yes	12(6.86)	136(4.53)			0.09	0.01
Self-reported history of gallbladder disease	No	155(88.57)	2864(95.34)	15.85**	<0.001	0.21	0.06
	Yes	20(11.43)	140(4.66)			0.21	0.06

*P<0.05 ** P<0.001

Appendix S1. (**Continued**) Characteristics of disease history covariates and standardized bias before and after ps adjusted using weighting by the odds in 20% of the total respondents, a cross sectional study in five cities, china, 2007-2008 (n=3,179)

		With FAB (%, n=175)	Without FAB (%, n=3004)	Chi square	P value	Standardized bias before PS adjusted	Standardized bias after PS adjusted using weighting
Self-reported history of Irritable Bowel Syndrome	No	172(98.29)	2999(99.83)	.*	0.008	0.12	0.11
	Yes	3(1.71)	5(0.17)			0.12	0.11
Self-reported history of Inflammatory bowel disease	No	169(96.57)	2957(98.44)	2.46	0.117	0.1	0.02
	Yes	6(3.43)	47(1.56)			0.1	0.02
Self-reported history of Esophageal or gastric cancer	No	175(100.00)	3002(99.93)	.	1.000	0	0
	Yes	0(0.00)	2(0.07)			0	0
Self-reported history of other gastrointestinal tumors	No	175(100.00)	3002(99.93)	.	1.000	0	0
	Yes	0(0.00)	2(0.07)			0	0
Self-reported history of recurrent pneumonia	No	175(100.00)	2991(99.57)	.	1.000	0	0
	Yes	0(0.00)	13(0.43)			0	0
Self-reported history of chronic pharyngitis	No	144(82.29)	2716(90.41)	12.10**	<0.001	0.21	0.03
	Yes	31(17.71)	288(9.59)			0.21	0.03
Self-reported history of chronic hoarseness	No	166(94.86)	2975(99.03)	21.03**	<0.001	0.19	0.09
	Yes	9(5.14)	29(0.97)			0.19	0.09
Self-reported history of chronic cough	No	162(92.57)	2959(98.50)	29.24**	<0.001	0.23	0.07
	Yes	13(7.43)	45(1.50)			0.23	0.07
Self-reported history of non-cardiac chest pain	No	172(98.29)	2986(99.40)	1.66	0.197	0.09	0.04
	Yes	3(1.71)	18(0.60)			0.09	0.04
Self-reported history of eczema	No	166(94.86)	2933(97.64)	4.14*	0.042	0.13	0.04
	Yes	9(5.14)	71(2.36)			0.13	0.04

*P<0.05 ** P<0.001
